# Targeting SR Proteins Improves SMN Expression in Spinal Muscular Atrophy Cells

**DOI:** 10.1371/journal.pone.0115205

**Published:** 2014-12-15

**Authors:** Claribel D. Wee, Mallory A. Havens, Francine M. Jodelka, Michelle L. Hastings

**Affiliations:** Department of Cell Biology and Anatomy, The Chicago Medical School, Rosalind Franklin University of Medicine and Science, North Chicago, Illinois, United States of America; Florida Atlantic University, United States of America

## Abstract

Spinal muscular atrophy (SMA) is one of the most common inherited causes of pediatric mortality. SMA is caused by deletions or mutations in the survival of motor neuron 1 (*SMN1*) gene, which results in SMN protein deficiency. Humans have a centromeric copy of the survival of motor neuron gene, *SMN2*, which is nearly identical to *SMN1.* However, *SMN2* cannot compensate for the loss of *SMN1* because *SMN2* has a single-nucleotide difference in exon 7, which negatively affects splicing of the exon. As a result, most mRNA produced from *SMN2* lacks exon 7. *SMN2* mRNA lacking exon 7 encodes a truncated protein with reduced functionality. Improving *SMN2* exon 7 inclusion is a goal of many SMA therapeutic strategies. The identification of regulators of exon 7 inclusion may provide additional therapeutic targets or improve the design of existing strategies. Although a number of regulators of exon 7 inclusion have been identified, the function of most splicing proteins in exon 7 inclusion is unknown. Here, we test the role of SR proteins and hnRNP proteins in *SMN2* exon 7 inclusion. Knockdown and overexpression studies reveal that SRSF1, SRSF2, SRSF3, SRSF4, SRSF5, SRSF6, SRSF7, SRSF11, hnRNPA1/B1 and hnRNP U can inhibit exon 7 inclusion. Depletion of two of the most potent inhibitors of exon 7 inclusion, SRSF2 or SRSF3, in cell lines derived from SMA patients, increased *SMN2* exon 7 inclusion and SMN protein. Our results identify novel regulators of *SMN2* exon 7 inclusion, revealing potential targets for SMA therapeutics.

## Introduction

Spinal muscular atrophy (SMA) is a pediatric neurodegenerative disorder that affects motor neurons and results in weakness and wasting of the voluntary muscles in the arms and legs. SMA has an incidence of 1 in 6000 live births and a carrier frequency of 1 in 40 and, in its most severe forms, causes death in the early years of life [1]. The disease is caused by deletion or mutation of the survival of motor neuron 1 (*SMN1*) gene that results in the insufficient production of SMN protein [2], [3]. All patients retain the centromeric *SMN2* gene, which also codes for SMN protein [4]. However, due to a single nucleotide difference at position +6 of *SMN2* exon 7, the exon is alternatively spliced and the majority of the mRNAs lack exon 7, resulting in a transcript that codes for a truncated and unstable form of SMN protein [5], [6]. An estimated 10% of the transcripts produced from an *SMN2* gene encode full-length protein derived from mRNA that includes exon 7. The amount of full-length protein produced from *SMN2* is an important determinant of disease severity. Individuals with more than two copies of *SMN2*, as a result of duplication, tend to have less severe forms of SMA [3], [7]. The correlation between *SMN2* expression and disease outcome suggests that increasing *SMN2* exon 7 inclusion to increase SMN protein abundance will be therapeutic. Although there is currently no FDA approved disease-modifying treatment available to patients, a number of therapeutic strategies aimed at improving exon 7 inclusion have demonstrated efficacy in animal models of the disease [8]–[13] and in early clinical trials in humans. Given the potential of splice-modulating approaches as a treatment for SMA, there is a need for a comprehensive understanding of the regulators of exon 7 inclusion in order to identify putative targets for therapeutics and to elucidate the mechanism of action of current therapeutic programs that target splicing.

Pre-mRNA splicing is directed by the spliceosome complex, which is comprised of five small nuclear ribonucleoprotein (snRNP) particles and additional proteins known as splicing proteins/factors [14]–[20]. The spliceosome identifies exons and introns by binding to consensus splicing sequences at the 5′ and 3′ ends of an intron. The recognition and binding of so-called 5′ and 3′ splice sites is aided by cis-acting splicing enhancer and splicing silencer elements within exons and introns. Enhancers and silencers are bound by splicing proteins that help to recruit or block the spliceosome. Alternative splicing arises in part through competition between mutually exclusive splice sites. Splice site competition can be affected by splicing silencers and enhancers and their cognate binding proteins.

A number of proteins and cis-acting sequence elements have been shown to regulate the alternative splicing of *SMN* exon 7 [21]. The serine/arginine (SR) splicing factor protein family is made up of twelve members [22] that play a role in both constitutive [23], [24] and alternative splicing [25]–[30]. Three of these proteins, SRSF1, SRSF2 and SRSF9 have been shown to influence exon 7 inclusion [31]–[34]. SRSF1 binds to exon 7 in *SMN1* to promote exon 7 inclusion, however, the C-T transition within exon 7 of *SMN2* disrupts this exonic splicing enhancer motif, contributing to the low level of *SMN2* exon 7 inclusion [35], [36]. SRSF2 activates exon 7 skipping but not inclusion in cell-free splicing assays [34]. SRSF9 interacts with hTra2-β1, a SR-like protein and known regulator of exon 7 splicing [37], and promotes exon 7 inclusion [33]. However, the roles of most members of the SR protein family in *SMN2* exon 7 splicing have not been explored. Likewise, there is a precedent to further investigate the members of the heterogenenous ribonucleoprotein (hnRNP) family with regard to *SMN2* exon 7 splicing. A number of hnRNP proteins have been shown to inhibit exon 7 inclusion, including hnRNP A1 [38], [39], hnRNP A2B1 [38], hnRNP C [40] and hnRNP U [41]. Conversely, hnRNP G appears to be an activator of exon 7 inclusion [42]–[44]. Clearly there is a complex interplay of splicing proteins contributing to the regulation of alternative splicing of *SMN2* exon 7. The dynamic and regulated expression of these splicing factors likely influences the splicing outcome. However, it is difficult to predict outcomes without a more comprehensive view of the regulators.

In the current study, we analyzed the activity of SR and hnRNP proteins in the context of *SMN2* splicing in order to further characterize the regulatory roles these proteins may play in SMA disease pathogenesis and to identify potential targets for therapeutics. We depleted or overexpressed SR and hnRNP proteins in cells and found that most of the proteins in the SR protein family and hnRNP A2/B1 and hnRNP U inhibit exon 7 inclusion. Depletion of SRSF2 and 3 in SMA patient-derived cells resulted in an increase in exon 7 inclusion and SMN protein abundance. Our results identify novel regulators of SMN2 exon 7 inclusion that could be targeted for the improvement of SMN expression in SMA cells.

## Materials and Methods

### Expression plasmids

The expression vectors, pCGT7-SRSF1, pCGT7-SRSF2, pCGT7-SRSF3, pCGT7-SRSF7, pCGT7-SRSF9, pFLAG-SRSF11 and pFLAG-GFP have been previously described [45]–[47].

### Primers and RNAi

Primer and siRNA sequences are provided in S1 Table.

### Cell culture and transfection

HeLa cells were maintained in Dulbecco’s modified Eagle medium (DMEM) supplemented with 10% (v/v) fetal bovine serum (FBS). Cells were plated at a density of 3.5×10^5^ cells per well onto six-well dishes 24 h prior to treatment. For treatment with RNAi, 50 nM duplex siRNA was transfected using Lipofectamine 2000 (Invitrogen) as per manufacturer’s instructions. Scrambled AllStars siRNA (Qiagen) was used as a control. Cells were grown for 48 h post transfection, at which point an additional treatment of 50 nM siRNA was given. Cells were then split 1∶2, 24 h after the second RNAi treatment and total RNA and protein were collected 24 h later. For overexpression experiments, 1 µg of expression vector was transfected using Lipofectamine 2000 (Invitrogen) as per the manufacturer’s instructions. Cells were split 1∶2, 24 h post-transfection and total RNA and protein were collected after an additional 24 h.

The human fibroblast cell line derived from a type I SMA patient with one copy of *SMN2* (GM00232; Coriell Cell Repository) was maintained in DMEM supplemented with 10% (v/v) FBS. SMA iPS cells were maintained as neural stem cells in neural progenitor growth medium (Stemline, Sigma) prior to transfection and then transferred to DMEM with 10% (v/v) fetal bovine serum (FBS) for transfections [48]. Cells were transfected with 50 nM siRNA using Lipofectamine 2000 (Invitrogen) as per manufacturer’s instructions. Scrambled AllStars siRNA (Qiagen) was used as a control. Treatment with 50 nM siRNA was repeated 48 h and 96 h after initial transfection and RNA and protein were collected after an additional 24 to 48 h.

### RNA extraction and RT-PCR

Prior to collection cells were washed with 1X PBS. Total RNA was isolated using TRIzol reagent (Invitrogen) and subsequently converted to cDNA using GoScript Reverse Transcription System (Promega). PCR was conducted with GoTaq Green Master mix (Promega). Reactions contained [α−^32^P]dCTP. *SMN1* and *SMN2* PCR products were digested with the restriction enzyme DdeI for 1 h at 37°C. Products were separated using 6% native polyacrylamide gel electrophoresis. Quantification of products was based on phosphorimage analysis on a Typhoon 9400 and ImageQuant T software (GE Healthcare). Calculations of transcript isoform percentages were normalized for cytosine content.

### Protein extraction and immunoblot

Cells were lysed with Laemmli buffer and heated at 100°C for 10 min. Protein samples were separated by sodium dodecyl sulfate (SDS)-PAGE and transferred to Immobilon-FL membrane (Millipore). Membranes were probed with a goat polyclonal antibody specific for bacteriophage T7 gene 10 tag (Novus), a mouse monoclonal antibody specific for the FLAG tag (Sigma), a mouse monoclonal antibody specific for mouse or human SMN (BD Biosciences), a mouse monoclonal antibody specific for SRSF1 [49], a mouse monoclonal antibody specific for SRSF2 (Millipore), a mouse monoclonal antibody specific for SRSF3 (Novus Biologicals), a rabbit polyclonal antibody specific for SRSF4 (Millipore), a goat polyclonal antibody specific for SRSF5 (Santa Cruz Biotechnology), a mouse monoclonal antibody specific for SRSF6 [50], a goat polyclonal antibody specific for SRSF11 (Santa Cruz Biotechnology), a mouse monoclonal antibody specific for β-catenin (BD Transduction Laboratories), or a mouse monoclonal antibody specific for β-actin (Sigma), followed by Alexa Fluor 594-conjugated anti-goat, anti-mouse, or anti-rabbit secondary antibody (Invitrogen) or horseradish peroxidase (HRP)-conjugated donkey anti-goat, goat anti-mouse, or goat anti-rabbit secondary antibody (Thermo). Detection and quantitative analysis of fluorescence was performed using a Typhoon 9400 and the ImageQuant T software package (GE Healthcare). Detection of HRP-labeled membranes was performed with either Luminata Classico Western HRP Substrate or Luminata Forte Western HRP Substrate (Millipore) and quantitation was preformed using NIH ImageJ software.

### In vitro transcription and cell-free splicing

DNA templates for *in vitro* transcription were generated from SalI digestion of pCI-SMN1 and pCI-SMN2 containing plasmids [31], [34]. RNA splicing substrates were transcribed using T7 RNA polymerase (Promega), transcription buffer (Promega), with 10 mM DTT, 0.5 mM A, C, 0.25 mM G, 0.01 mM or 0.25 mM U, α−^32^P UTP, RNase inhibitor (Promega), and 0.5 µM 7Me-GpppG cap analog (New England Biolabs). The reactions were incubated for 1 h at 37°C and subsequently treated with RQ1 DNase (Promega) for 30 min at 37°C. Reaction products were separated on 5% denaturing PAGE gels, extracted, eluted, ethanol precipitated and reconstituted in water prior to use.

*In vitro* transcribed RNA (5–10 fmol) was combined with HeLa nuclear extract under splicing conditions (32 mM HEPES, 2 mM MgCl_2_, 1.95% polyvinyl alcohol, 1X buffer D (20 mm HEPES-KOH, pH 8; 100 mm KCl; 0.2 mm EDTA; 20% (v/v) glycerol), 60 mM KCl, 0.5 mM ATP and 20 mM creatine phosphate) with or without additional SR proteins prepared as previously described [51]. The reactions were incubated for 2 hrs at 30°C. Reactions were stopped with stop buffer (0.3 M sodium acetate and 0.1% w/v SDS) and RNA was phenol extracted and precipitated with ethanol. RNA was reverse transcribed using a reverse primer to exon 8 using Goscript RT (Promega), as per manufacturer’s instructions. PCR of the cDNA was performed with a forward primer specific to exon 6 and the reverse primer to exon 8 using GoTaq Green master mix (Promega).

### Statistics

Statistical significance was determined using the Student’s or one sample *t* test as detailed in Figure legends.

## Results

### Differential effects of SR proteins on *SMN2* exon 7 inclusion in cell-free splicing assays

**Figure 1:**
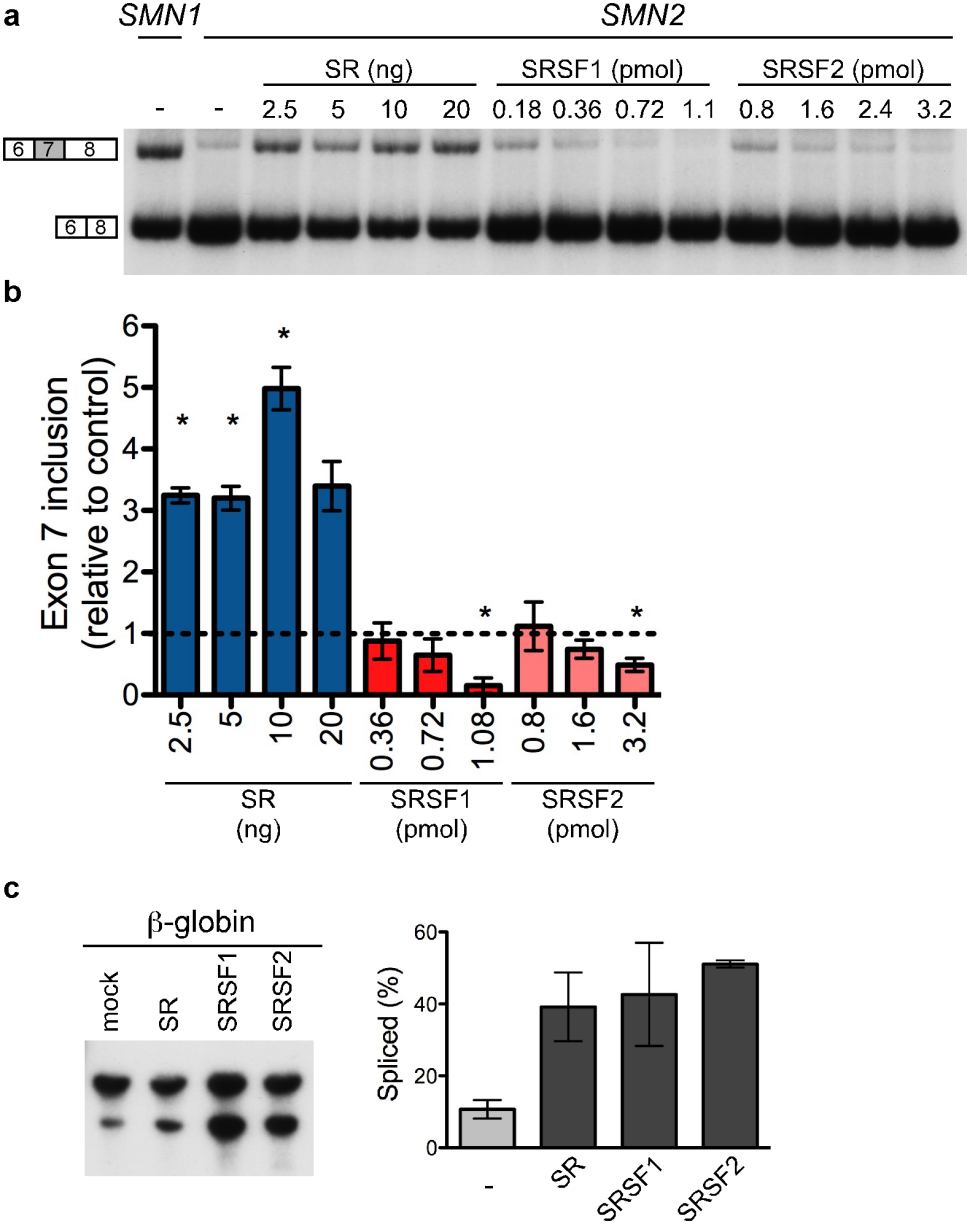
SR proteins regulate *SMN2* exon 7 splicing in a cell-free splicing assay. (**A**) Semi-quantitative radiolabeled RT-PCR analysis of *in vitro* transcribed *SMN1* and *SMN2* RNA isolated from cell-free splicing reactions with or without the addition of the indicated recombinant SR proteins or a cellular fraction enriched in SR proteins (total SR). (**B**) Quantification of *SMN2* exon 7 splicing following addition of the indicated SR proteins. Splicing is normalized to *SMN2* exon 7 splicing in nuclear extract without additional SR protein (dashed line indicates basal exon 7 splicing). Only experiments repeated more than once are included in the graph. Error bars represent the standard error of the mean (SEM); total SR (2.5 ng n = 2, 5 ng n = 4, 10 ng n = 4), SRSF1 (0.36 pmol n = 4, 0.72 pmol n = 4, 1.08 pmol n = 2), SRSF2 (0.8 pmol n = 2, 1.6 pmol n = 4, 3.2 pmol n = 4). Asterisks indicate p value (two-tailed) ≤0.05 as determined by a one sample t test with a theoretical value of 1.0 for normalization to basal *SMN2* exon 7 splicing. (**C**) Analysis of *in vitro* transcribed β-globin RNA splicing in a cell-free splicing reaction with or without (–) the addition of total SR proteins (10 ng), SRSF1 (0.72 pmol) or SRSF2 (1.6 pmol) to verify recombinant protein activity. The graph shows the percent of RNA that is spliced: (spliced/(unspliced+spliced)*100), error bars are SEM, n = 2.

Improvement of SMN2 exon 7 splicing is a goal of many SMA therapies. SR proteins are a family of splicing factors known for their role in the enhancement of splicing, and thus may be potential targets for SMA therapeutic strategies. In order to test whether SR proteins can directly affect *SMN2* exon 7 inclusion, we performed *in vitro*, cell-free splicing assays of exon 7 splicing in HeLa nuclear extracts supplemented with a cell fraction enriched in SR proteins [51], [52]. We found, not unexpectedly, that SR proteins increase exon 7 inclusion (Fig. 1). In order to identify the individual SR proteins that may be promoting exon 7 inclusion, we supplemented HeLa extract with individual recombinant SR proteins and found that different SR proteins had protein-specific effects on exon 7 splicing (Fig. 1). Specifically, addition of purified, recombinant SRSF1 or SRSF2 reduced exon 7 inclusion, the opposite effect of the total SR protein fraction (Fig. 1A). The functionality of the SR proteins in the *in vitro* reactions was verified by testing them in the splicing of a constitutively spliced β-globin intron [24], [51] (Fig. 1B), the splicing of which has been shown previously to be enhanced by these SR proteins [23], [24]. These results indicate that, although a combination of SR proteins can promote exon 7 inclusion, individual SR proteins can inhibit splicing of the exon.

### Overexpression of a subset of SR proteins reduces *SMN2* exon 7 inclusion

**Figure 2:**
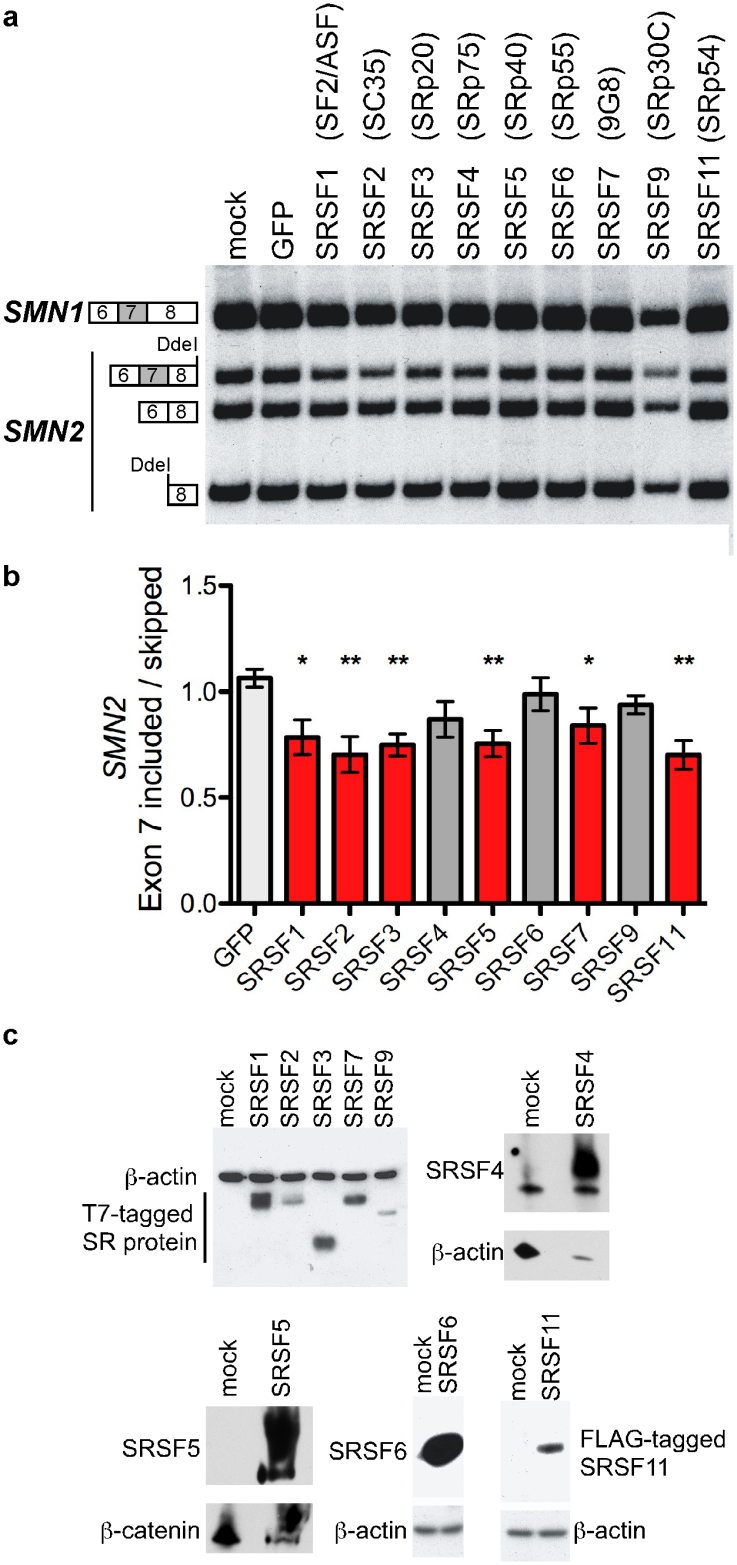
Overexpression of SR proteins modulates endogenous *SMN2* exon 7 inclusion in cells. (**A**) Semi-quantitative radiolabelled RT-PCR of endogenous *SMN2* mRNA following selective over-expression of SR proteins in HeLa cells. Reaction products were digested with DdeI to distinguish between *SMN1* and *SMN2* transcripts, digestion products are indicated on the left. Mock samples were exposed to the transfection reagent in the absence of siRNA. (**B**) The graph represents quantification of *SMN2* exon 7 inclusion: (Exon 7 included/skipped). Asterisks indicate a statistically significant decrease in exon 7 inclusion p≤0.05 and **p≤0.01, by unpaired Student’s t tests. Error bars represent SEM. In all cases, SRSF1, SRSF2, SRSF3, SRSF4, SRSF5, SRSF6, SRSF7, SRSF9 n = 6; SRSF11 n = 5. (**C**) Immunoblot analysis of protein lysates from HeLa cells transfected with the indicated expression vectors. Immunoblots of T7-tagged SRSF1, SRSF2, SRSF3, SRSF7, and SRSF9 probed with a T7-specific antibody, endogenous SRSF4, 5 and 6 probed with SRSF4-, SRSF5- and SRSF6-specific antibodies and FLAG-tagged SRSF11 probed with a FLAG-specific antibody. β-actin and β-catenin were analyzed for loading control.

To define the activity of individual SR proteins in *SMN2* exon 7 splicing, we overexpressed SR proteins in cells (Fig. 2). SRSF1, 2, 3, 4, 5, 6, 7, 9, or 11 expression vectors were transfected into HeLa cells and the effect of overexpression of these proteins on exon 7 splicing was analyzed by RT-PCR. To distinguish *SMN1*-derived RNA transcripts from *SMN2,* PCR products were digested with the restriction endonuclease *DdeI*, which cleaves within exon 8 of *SMN2*-derived, but not *SMN1*-derived transcripts (Fig. 2A, B). Overexpression of the proteins was verified by immunoblot analysis (Fig. 2C). SRSF1, 2, 3, 5, 7 or 11 overexpression caused a significant decrease of *SMN2* exon 7 inclusion when compared to overexpression of the control vector, GFP (Fig. 2A, B). The lack of a significant effect of overexpression of SRSF4, 6, and 9 on exon 7 splicing suggests either that these proteins do not have a role in exon 7 splicing, that the level of overexpression is not sufficient to elicit an effect, or that the abundance of endogenous protein is sufficient to exert a maximal effect on splicing that cannot be further enhanced by higher protein expression in cells. Secondary effects of protein overexpression may also influence the outcome of exon 7 splicing. In any case, these data show that SRSF1, 2, 3, 5, 7 and 11 can regulate *SMN2* exon 7 inclusion and that overexpression of these proteins induces exon 7 skipping in cells either directly or indirectly.

### Cellular reduction of select SR and hnRNP proteins improves *SMN2* exon 7 inclusion

**Figure 3:**
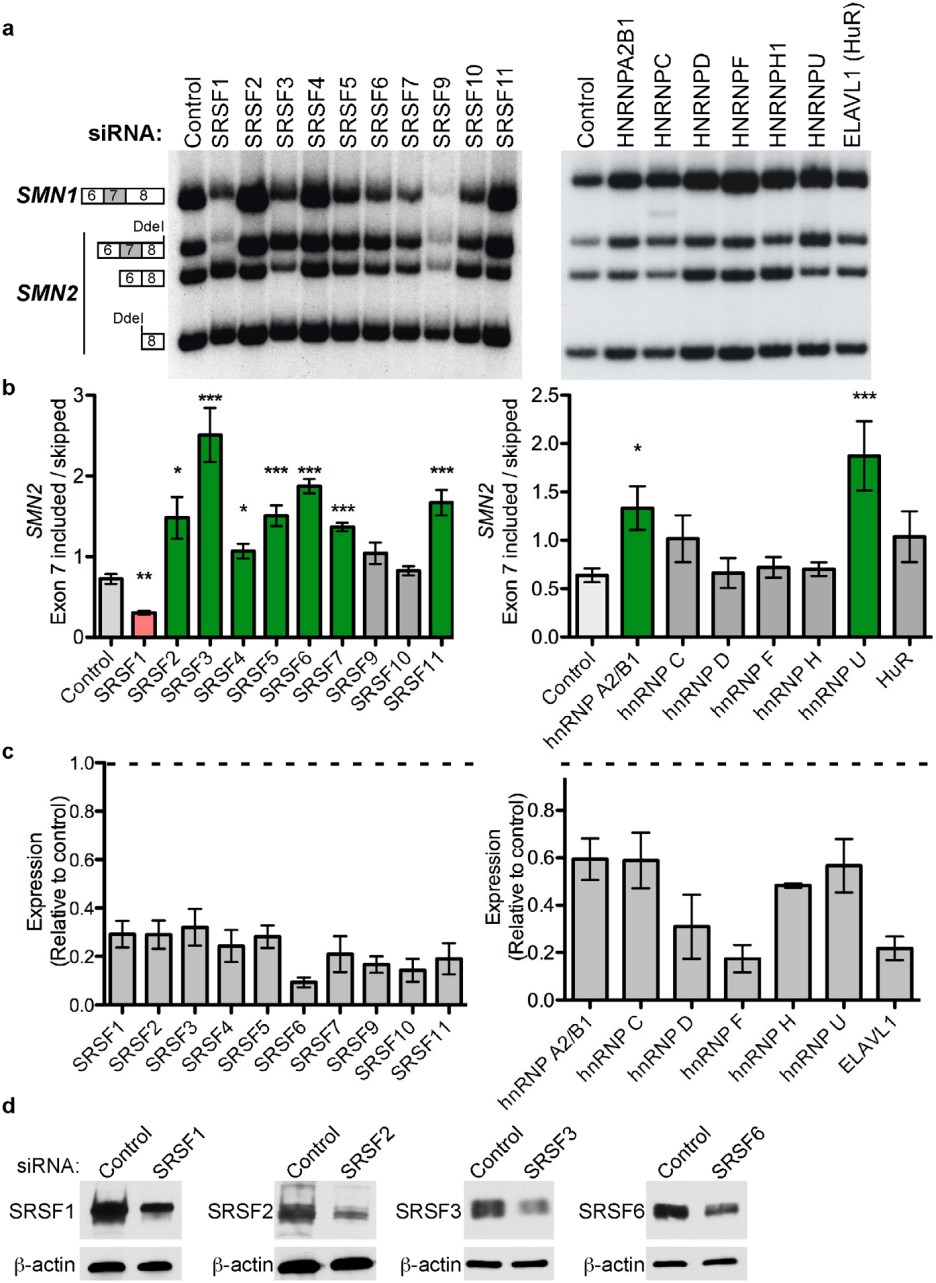
Knockdown of SR and hnRNP proteins modulates endogenous *SMN2* exon 7 inclusion. (**A**) Semi-quantitative radiolabelled RT-PCR of endogenous *SMN2* mRNA following RNAi-mediated knockdown of individual SR or hnRNP proteins in HeLa cells. Reaction products were digested with DdeI. Digestion products are labeled. (**B**) Graphs show the quantification of *SMN2* exon 7 inclusion: (included/skipped). Asterisks indicate a statistically significant change in exon 7 inclusion, *p≤0.05, **p≤0.01 and ***p≤0.001, unpaired Student’s T-tests. (**C**) Quantification of RT-PCR confirmed effective, selective knockdown of SR proteins and hnRNPs. The dashed line represents the level of control expression. In all cases, error bars represent SEM. For SRSF1 and SRSF2 n = 3; SRSF4, SRSF6, hnRNP A2/B1, ELAVL1 and hnRNP D n = 4; SRSF7, SRSF10, and hnRNP U n = 5; SRSF9, hnRNP C, hnRNP H1 and hnRNP F n = 6, SRSF5 n = 7, SRSF3 and SRSF11 n = 8; Control cells were transfected with a scrambled control siRNA. (**D**) Immunoblot analysis of indicated endogenous protein from lysates of cells treated with siRNAs targeting the indicated SR protein or a non-specific control siRNA. β-actin is a loading control.

Alternative splicing of an exon is predicted to change when the abundance of one of its splicing regulators increases or decreases. Having seen effects on the splicing of *SMN2* exon 7 with the overexpression of SR proteins, we next knocked down each of the 12 SR proteins in the SR family of splicing factors using siRNAs in HeLa cells (Fig. 3A, B). Expression of ten of the twelve SR proteins was depleted by more than 60% (Fig. 3C, D). We were not successful in depleting SRSF8 or SRSF12 from cells. Individual knockdown of seven of the ten SR proteins, SRSF2, 3, 4, 5, 6, 7, and 11, caused a significant increase in exon 7 inclusion (Fig. 3A, B). SRSF1 was the only SR protein knockdown that caused a significant decrease of *SMN2* exon 7 inclusion (Fig. 3A, B), which may reflect an indirect regulation of exon 7 splicing in cells by SRSF1. Reduction of SRSF9 and SRSF10 expression did not cause a statistically significant change in *SMN2* exon 7 inclusion following knockdown. Taken together with the overexpression assays (Fig. 2), these results reveal SRSF2, 3, 4, 5, 6, 7, and 11 as previously unknown negative regulators of *SMN2* exon 7 inclusion in cells.

Although our results suggest that many SR proteins inhibit exon 7 inclusion, SR proteins are commonly known for their role in the enhancement of splicing [53]. In contrast, the hnRNP family of RNA binding proteins, are frequently implicated in the inhibition of splicing [54]. In order to test the role of hnRNP proteins in exon 7 splicing, we depleted a number of hnRNP proteins from HeLa cells using siRNAs. A 40–50% reduction in hnRNP A2B1 and hnRNP U mRNA levels (Fig. 3C) led to a significant increase in *SMN2* exon 7 inclusion, whereas knockdown of hnRNP C, D, F, H or ELAVL1 had no significant effect on splicing (Fig. 3A, B). These results confirm hnRNP A2B1 and hnRNP U are negative regulators of *SMN2* exon 7 inclusion as shown in previous studies [38], [40], [41] and demonstrate that a number of other hnRNP proteins do not appear to have a significant role in the regulation of exon 7 inclusion.

### SR protein knockdown in SMA patient-derived cells increases *SMN2* splicing and protein abundance

**Figure 4:**
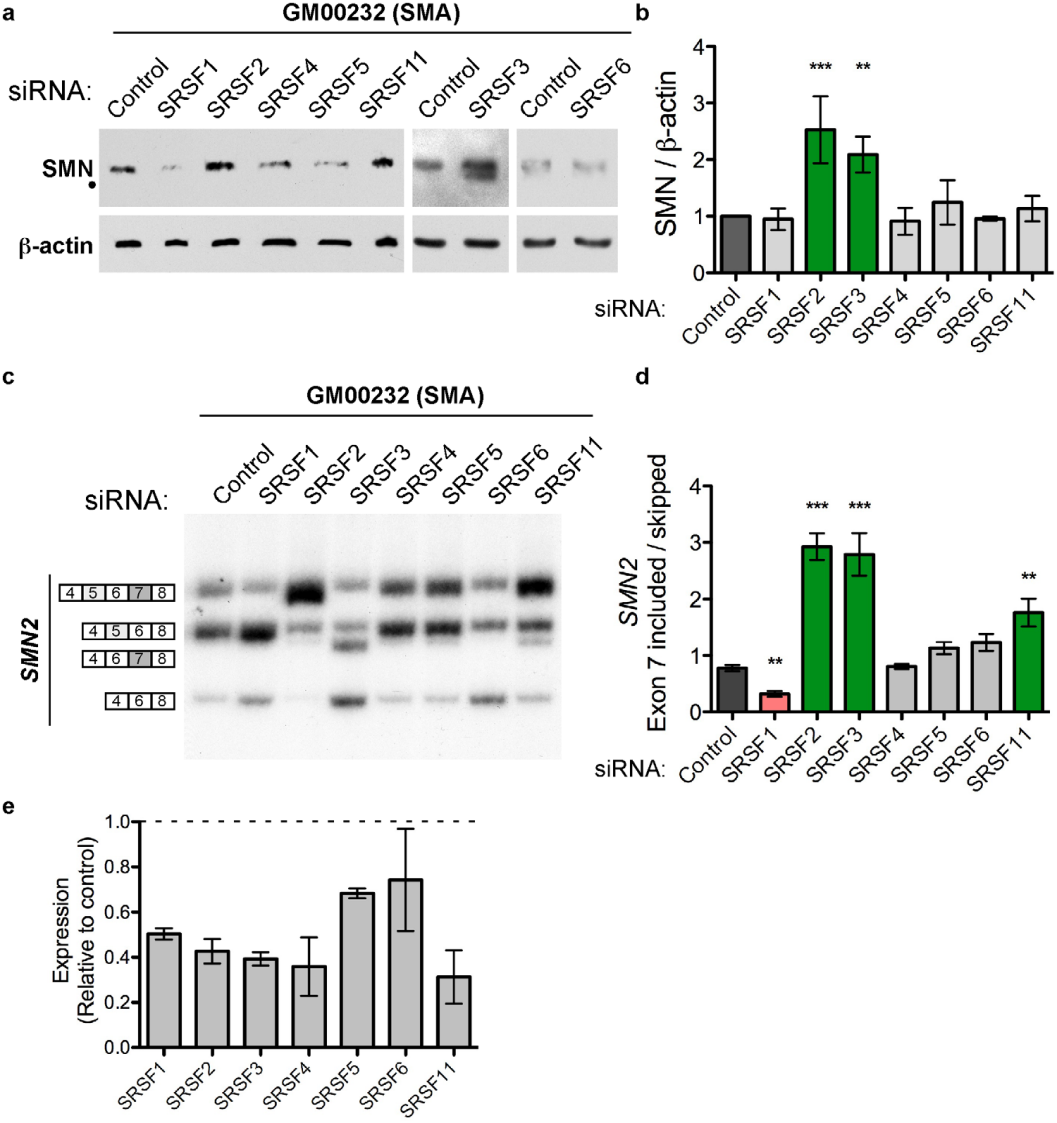
Knockdown of SRSF2 and SRSF3 in SMA cells increases *SMN2* exon 7 inclusion and SMN protein abundance. (**A**) Immunoblots of SMN and β-actin following knockdown of SR proteins in GM00232 type 1 SMA patient-derived fibroblast cell line. • indicates a putative SMN protein lacking exon 5. (**B**) Quantification of protein abundance: (SMN/β-actin). (**C**) Semi-quantitative radiolabelled RT-PCR showing endogenous *SMN2* exon 7 inclusion following knockdown of SR proteins in GM00232 cells. (**D**) Quantification of *SMN2* exon 7 inclusion (included_All isoforms including exon 7_/skipped_All isoforms skipping exon 7_) corrected for cytosine content. (E) Quantitation of RT-PCR experiments to assess mRNA expression of indicated SR proteins following siRNA treatment. The dashed line represents the level of expression in control-treated cells. Error bars represent SEM. SRSF1, SRSF4, SRSF5 and SRSF6 n = 3, SRSF2 and SRSF11 n = 4, SRSF3 n = 5. Asterisks indicates a statistically significant change in SMN abundance or *SMN2* exon 7 inclusion, *p≤0.05, **p≤0.01 and ***p≤0.001, unpaired Student’s T-tests. Control cells were transfected with a scrambled control siRNA.

We next tested the role of SR proteins in *SMN2* splicing in a type 1 SMA patient-derived fibroblast cell line (GM00232), which allowed us to further assess the potential relevance of these splicing factors as therapeutic targets in a context that is more pertinent to disease physiology. The SMA patient-derived fibroblasts are a good peripheral cell model for SMA as recent work has shown that restoration of *SMN2* exon 7 inclusion outside of the nervous system may be an important therapeutic goal in addition to restoration within the nervous system [10]. We knocked down SRSF1, 2, 3, 4, 5, 6 and 11 in the SMA fibroblast cells and assessed the effect on SMN protein abundance as well as *SMN2* mRNA splicing (Fig. 4). Knockdown of SRSF2 or SRSF3 resulted in an increase in SMN protein, demonstrating that down-regulation of these SR proteins can increase SMN protein abundance.

Knockdown of SRSF3 in the SMA patient-derived cells produced a smaller isoform of SMN protein in addition to the full-length protein (•, Fig. 4). In humans, an alternatively spliced form of *SMN1* lacking exon 5 (Δ5) is a common isoform, though the function of the protein isoform is not clear [55], [56]. Therefore, we analyzed both exon 5 and exon 7 splicing following SR protein knockdown in the SMA patient fibroblast cell line (Fig. 4C, D). Individual knockdown of SRSF2, SRSF3 and SRSF11 resulted in an increase in exon 7 inclusion, and knockdown of SRSF1 caused a decrease in exon 7 inclusion, consistent with our results in HeLa cells (Fig. 4C, D, E). Knockdown of SRSF3, and to a lesser extent SRSF11, also resulted in a significant decrease in mRNA transcripts that exclude exon 5. These data confirm that these four SR proteins modulate *SMN2* alternative splicing in cellular models that recapitulate SMA disease physiology. Furthermore, SRSF3 is both a positive regulator of *SMN2* exon 5 splicing and a negative regulator of exon 7 inclusion.

Knockdown of the other SR proteins did not result in more SMN2 exon 7 splicing or an increase in SMN protein abundance (Fig. 4A, B). However, knockdown of SRSF5 and 6 was less efficient than knockdown of the other SR protein in these patient derived cells (Fig. 4E) and less efficient than the knock-down in Hela cells (Fig. 3), making it difficult to conclude that these proteins do not have an effect on exon 7 splicing in these patient-derived cells.

**Figure 5:**
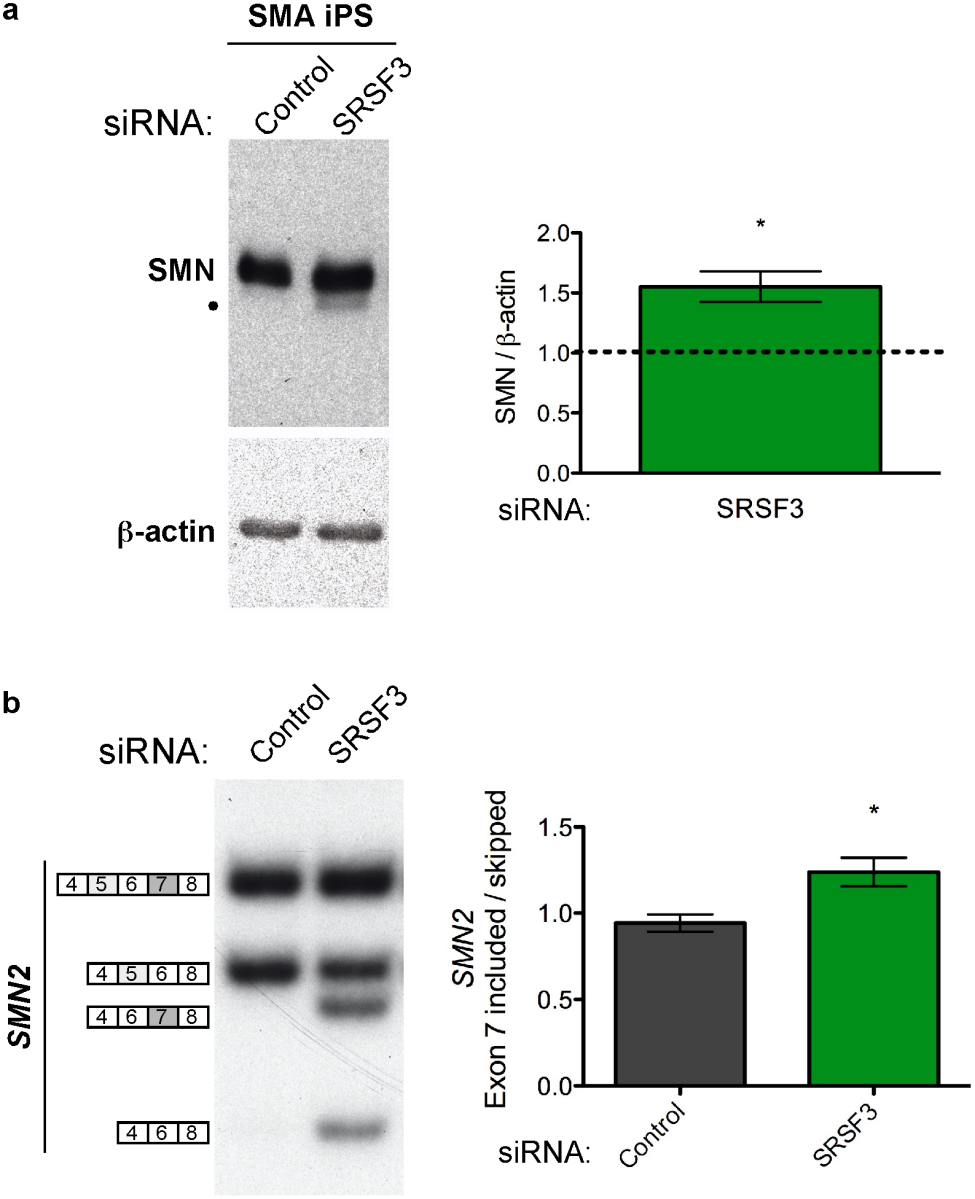
SRSF3 regulates *SMN2* expression in SMA patient iPS cells. (**A**) Immunoblot of SMN and β-actin following knockdown of SRSF3 with siRNA in SMA patient-derived iPS cells. • indicates a putative SMN protein lacking exon 5. Quantitation is shown on the right (SMN/β-actin). Asterisks indicate p value (two-tailed) ≤0.05 as determined by a one sample t test with a theoretical value of 1.0 for normalization to SMN in control-treated cells. The dashed line represents the level of expression in control-treated cells. (**B**) Semi-quantitative radiolabelled RT-PCR of endogenous *SMN2* mRNA after knockdown of SRSF3 using in SMA patient iPS cells or a scrambled siRNA (Control). Products are indicated on the left. Quantification of *SMN2* exon 7 inclusion (inclusion/skipped). Error bars represent SEM, n = 3. Asterisk represents a statistically significant increase in exon 7 inclusion where p≤0.05, unpaired Student’s T-tests.

We next tested the effect of knockdown of the most effective inhibitor of exon 7 inclusion, SRSF3, in multipotent neural stem cells generated from iPS cells derived from a type 1 SMA patient [48], [57], [58]. Similar to the results in the SMA patient fibroblasts, SRSF3 knockdown in iPS cells resulted in an increase in SMN protein abundance (Fig. 5A). This increase in SMN presumably results from corresponding increase in exon 7 inclusion (Fig. 5B), which further demonstrates the role of SRSF3 as an inhibitor or exon 7 splicing. Because SMA is a neurodegenerative disease, the iPS cells used in this experiment may be one of the better *in vitro* cellular models for SMA and offer further proof of concept that targeted reduction of cellular SRSF3 would result in an increase in SMN protein in a cell type that is relevant for SMA.

## Discussion

In this study we tested members of the SR and hnRNP families of splicing factors for their roles in regulating *SMN2* exon 7 alternative splicing. We find that the majority of SR proteins, SRSF1, 2, 3, 4, 5, 6, 7, and 11, and two hnRNPs, hnRNP A2/B1 and U, are regulators of *SMN2* exon 7 inclusion as evidenced by their effect on splicing in a cell-free assay or when knocked down and/or overexpressed in cells. A decrease in the abundance of a number of other RNA binding proteins, SRSF9 and 10, and hnRNP C, D, F, H and ELAVL1 did not affect exon 7 inclusion. We demonstrate that lowering the abundance of two of the most potent inhibitors, SRSF2 and SRSF3, improved *SMN2* exon 7 inclusion and SMN protein abundance in SMA patient cell lines. Together, these findings reveal novel regulators of exon 7 inclusion and thereby, provide new potential therapeutic targets for the treatment of SMA.

**Figure 6:**
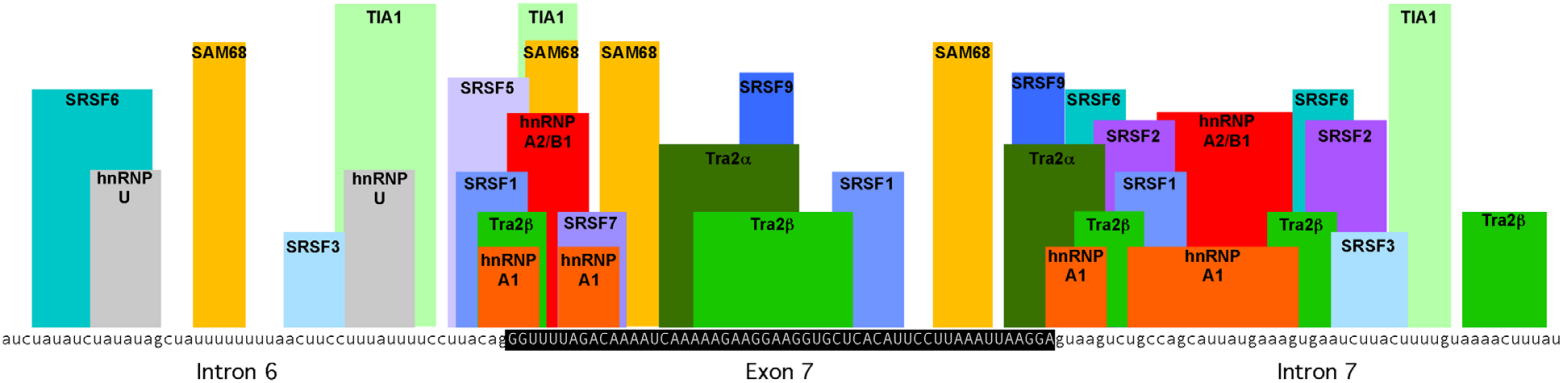
Splicing factors with experimentally validated affects on *SMN2* exon 7 inclusion are shown at their predicted binding sites. Exon 7 (capital letters within the black box) and 50 nucleotides of introns 6 and 7 (lower case letters) are shown. Binding motifs and references are provided in S1 Table.

Our study adds to the number of splicing factors that have been demonstrated to regulate *SMN2* exon 7 inclusion (Table 1). In order to show where known regulators of exon 7 inclusion may be acting on the *SMN2* pre-mRNA, we mapped predicted and experimentally validated binding sites of splicing factors that regulate *SMN2* exon 7 inclusion [31], [37], [41], [43], [59]–[62], including those identified in the current study, onto the pre-mRNA sequence containing *SMN2* exon 7, the 3′ end of intron 6, and the 5′ end of intron 7 (Fig. 6, S2 Table). This map acts as a general guide to the exon 7 regions that may contain cis-acting regulatory sequences and the trans-acting factors that may function to regulate exon 7 inclusion through binding to these sites. Extensive mutational analysis and ASO targeting strategies have confirmed sequences that are important for determining the inclusion of the exon [21], [63]–[65], many of which are consistent with the activity of the splicing factors that recognize these sequences. We have limited our analysis of regulatory sequences to the regions immediately flanking exon 7 (within 50 nucleotides). However, it should be noted that antisense oligonucleotides directed to sequences more distant from exon 7 have been identified that improve exon 7 inclusion [66]–[69]. Indeed, there have been numerous regulatory sequences and binding proteins mapped to more distant regions surrounding exon 7 [70], [71].

**Table 1:** Splicing factors tested for activity in *SMN2* exon 7 splicing.

Protein	Effect on exon 7 inclusion	Reference
SRSF1 (SF2/ASF)	enhancement/inhibition	current study [31], [32], [34]
SRSF2 (SC35)	inhibition	current study [32]–[34]
SRSF3 (SRp20)	inhibition	current study
SRSF4 (SRp75)	inhibition	current study
SRSF5 (SRp40)	inhibition	current study [33]
SRSF6 (SRp55)	inhibition	current study [33]
SRSF7 (9G8)	inhibition	current study [32], [33], [41]
SRSF9 (SRp30c)	Neutral/enhancement	current study [33]
SRSF10 (SRp38/FusIP1)	neutral	current study
SRSF11	inhibition	current study
hnRNP A1	inhibition	[38], [39], [41], [59]
hnRNP A2B1	inhibition	current study [38], [41]
hnRNP C	inhibition/enhancement	current study [40], [41]
hnRNP D	neutral	current study
hnRNP F	neutral	current study [41]
hnRNP G/RBMX	enhancement/neutral	[42], [44]
hnRNP H1	neutral	current study [59]
hnRNP K	neutral	[41]
hnRNP L	neutral	[41]
hnRNP M	neutral	[41]
hnRNP RALY	neutral	[41]
hnRNP Q	enhancement/inhibition	[83]
hnRNP U	inhibition	current study [41]
HuR/ELAVL1	weak inhibition	current study [60]
PSF	enhancement	[84]
Puf60	inhibition	[41], [85]
RBM10	inhibition	[41]
Sam68	inhibition	[86]
SF1	inhibition	[41]
SmB	enhancement	[58]
SmD	enhancement	[58]
SMN	enhancement	[58]
SON	inhibition	[41]
U1 snRNP	enhancement	[58]
U2 snRNP	enhancement	[58], [87]
U4 snRNP	enhancement	[58]
U5 snRNP	enhancement	[58]
U170K	enhancement	[58]
U2AF35	inhibition	[41], [58], [59], [87]
U2AF65	neutral	[41], [58], [87]
U2 B”	enhancement	[58]
TDP-43	enhancement	[88]
TIA1	enhancement	[61]
Tra2β	enhancement/neutral	[33], [37], [75]
ZIS2/ZNF265	inhibition	[89]

Prediction and identification of splicing factor binding sites (Fig. 6 and S2 Table) and ASO studies that indicate the presence of regulatory sequences, aid in understanding the regulation of exon 7 inclusion. It is likely that complex splicing protein interactions and cis-acting sequence elements, together, determine the outcome of splicing. Uncovering the mechanism of action of proteins in splicing is complicated by the potential for a particular protein to have multiple binding sites within a pre-mRNA. Also, binding of a particular protein at different sites, and in the context of other splicing proteins, may influence splicing in different ways. This type of multicomponent regulation may help to explain our observations that SRSF1 inhibited splicing in the cell-free splicing assay (Fig. 1) and when overexpressed in cells (Fig. 2), but also was required for splicing of the exon, as evidenced by the decrease in exon 7 inclusion when it is knocked down (Fig. 3). The change in *SMN2* exon 7 inclusion may reflect a change in the balance of SRSF1 at other binding sites that have conflicting affects on exon 7 inclusion. The effect of other SR proteins, many of which inhibit exon 7 inclusion (Fig. 2), may negatively influence exon 7 inclusion when SRSF1 is absent. Cooperative and competitive interactions between SR proteins are not uncommon and have been shown to regulate splicing in a complex manner [72]. Although regulation of *SMN2* exon 7 inclusion by some splicing factors may be both direct and indirect, the ability to regulate exon 7 inclusion makes the study of the factors relevant to SMA.

Overall, we have identified novel regulators of exon 7 inclusion, and also confirmed the activity of proteins that have been tested for exon 7 inclusion in other studies. One exception was with SRSF9. We did not observe a statistically significant change in exon 7 inclusion with knockdown or overexpression of SRSF9, which was previously reported to stimulate exon 7 inclusion via interactions with Tra2-β1 [33]. A minigene form of *SMN2* was used in the previous work, which may be more or less sensitive to changes in SR protein abundance than the endogenous transcript.

As the list of exon 7 splicing regulators grows, it is likely that many of the proteins do not directly affect splicing but rather alter other splicing or RNA-related processes that have an impact on splicing of exon 7. Indeed, several proteins likely regulate splicing via their role in U snRNP maturation, including hnRNP U [41], U1–70K, U2 B”, and Sm proteins [58] and SMN itself [58], [73]. Several other splicing factors have been shown to act indirectly via interactions with other proteins or possibly by influencing transcription [74], [75]. It is also possible that some of the proteins may have both direct and indirect affects on exon 7 inclusion, the combined effects of which may be cumulative or competitive.

Individual members of the SR family of proteins may be good therapeutic targets because previous studies suggest that there may be some level of functional redundancy among family members [45], [76]. Knockdown or sequestration of individual SR proteins to inhibit their interactions with their target sequences may prove to have less toxicity than other therapeutic strategies. Furthermore, we previously demonstrated that SMN functions in a feedback loop regulating its own expression [58]. Thus, it is possible that a modest down-regulation of single or multiple SR protein regulators of *SMN2* exon 7 inclusion would be sufficient to result in an improvement in full-length SMN protein that will be adequate to increase SMN expression to therapeutic levels, without fully disrupting other necessary functions of the splicing factors. In this way, subtle modulation of combinations of individual SR proteins could be therapeutic in SMA. This principle of combinatorial targets for disease therapy can be used to lower toxicity associated with targeting individual proteins, such as splicing factors, that have constitutive functions in the cell.

The identification of novel regulators of *SMN2* exon 7 inclusion is useful when evaluating drugs that have been shown to improve SMN protein expression. For example, we have reported on a tetracycline-like small molecule, PTK-01, that promotes exon 7 inclusion directly through the splicing reaction [9]. The mechanism by which PTK-01 acts is not known, however, it is possible that interactions with one or more positive or negative regulators of splicing may mediate the activity of the compound. The identification of regulators of exon 7 inclusion my also aid in the evaluation of the limitations of particular drug-candidates. For example, the small molecules valproic acid, sodium butyrate, or 5-(N-ethyl-N-isopropyl)-amiloride have been shown to increase the intracellular concentration of SRSF3 [77], [78]. It is possible that increasing negative regulators of exon 7 inclusion may compete with the desired effect of increasing full-length SMN protein expression, thereby decreasing drug efficacy. Likewise, the mechanism of antisense oligonucleotides (ASOs), which hold great promise as a therapeutic for SMA [10], can be better understood by a thorough understanding of the RNA binding proteins, such as the SR and hnRNP families of splicing factors, which may be competing for binding with ASOs [39].

In addition to identifying potential targets for manipulating the splicing of *SMN2* exon 7, our results also provide insight into possible mechanisms for tissue-specific alternative splicing of exon 7 which could account, in part, for the cell-type specific pathological affects caused by the loss of functional *SMN1* in SMA [73], [79]. Some cell-types may have greater *SMN2* exon 7 inclusion and thereby higher SMN protein expression, protecting them from deficits associated with SMN protein insufficiency. Indeed, expression of splicing factors varies widely between cell and tissue types and the interplay between these factors and their expression levels may regulate exon 7 inclusion [72], [80]–[82].

## Supporting Information

S1 Table **siRNA duplex and primer sequences used.**
(DOC)

S2 Table **Binding sites of proteins shown to affect** ***SMN2*** **exon 7 splicing.** ***SMN2*** **exon 7 and 50 nts of the upstream and downstream introns were considered for this table.** A depiction of the binding sites is shown in Fig. 6. **R** = G or A; **Y** = C or U; **W** = A or U; **N** = A, G, C or U; **K** = U or G; **S** = G or C; **D** = A, G or U; **M** = A or C; **H** = A, C or U; **V** = A, C or G; **B** = G, C or U.
(DOC)
