# Supplementary material for: Targeting SR Proteins Improves SMN Expression in Spinal Muscular Atrophy Cells
Source: PLoS One. 2014 Dec 15;9(12):e115205. doi: 10.1371/journal.pone.0115205 (PMC4266657; doi:10.1371/journal.pone.0115205)
Supplement: S1 Table — siRNA duplex and primer sequences used. (DOC) [file pone.0115205.s001.doc]

| **Gene** | **siRNA sequence** | **Primers** | |
| --- | --- | --- | --- |
| SRSF1 | 5'-GCAGAUGAACUCGGGAUG-3' | F | 5'-CAGAGTGGTTGTCTCTG-3' |
|  | 3'-CGUCUACUUGAGCCACUAC-5' | R | 5'-CTCCACGACACCAGTGCC-3' |
| SRSF2 | 5'-CCGCACUCGUUCUCGAUCUTT-3' | F | 5'-GGACGCCGGAGCCGCAG-3' |
|  | 3'-AGGGCGUGAGCAAGAGCUAGA-5' | R | 5'-GAGATCGAGAACGAGTGC-3' |
| SRSF3 | 5'-GCUAGAUGGAAGAACACUAT-3' | F | 5'-ATGCATCGTGATTCCTG-3' |
|  | 3'-CTCGAUCUACCUUCUUGUGAU-5' | R | 5'-CTGCGACGAGGTGGAGG-3' |
| SRSF4 | 5'-GGACUGCCUCCAAGUGGAATT-3' | F | 5'-GTTACGGTTCTGGACGC-3' |
|  | 3'-GACCUGACGGAGGUUCACCUU-5' | R | 5'-GCTCCGGGAGCGGGAG-3' |
| SRSF5 | 5'-CCUCGAAAUGAUAGACGAATT-3' | F | 5'-GATCCAAGGGATGCAGATG-3' |
|  | 3'-TTGGAGCUUUACUAUCUGCUU-5' | R | 5'-CTATCATTTCGAGGTCTGCG-3' |
| SRSF6 | 5'-GCAUAGGGUUGACUGAUAATT-3' | F | 5'-GTGGATACAGCAGTCGG-3' |
|  | 3'-CTCGUAUCCCAACUGACUAUU-5' | R | 5'-CTGGATCTGCTTCCAGAG-3' |
| SRSF7 | 5'-CGACGUCCCUUUGAUCCAATT-3' | F | 5'-GGTCTAGATCACATTCTCG-3' |
|  | 3'-GGGCUGCAGGGAAACUAGGUU-5' | R | 5'-CCAGACCTAGATCTTCTG-3' |
| SRSF8 | 5'-GGAUUACAUUGGAGCCAAUTT-3' | F | 5'-ATGAGGACAGGTGGCCG-3' |
|  | 3'-ATCCUAAUGUAACCUCGGUUA-5' | R | 5'-GCGCTGCACTGGGCGCTG-3' |
| SRSF9 | 5'-UGGUUAUGAUUAUGGCCAGTT-3' | F | 5'-GGCCAGTGTCGGCTTCGTG-3' |
|  | 3'-TTACCAAUACUAAUACCGGUC-5' | R | 5'-GGTGTCATCCAGTTTACGC-3' |
| SRSF10 | 5'-GGAUGUUCGUGAUGCUGAATT-3' | F | 5'-GTCTGAAGACTTGCGGCG-3' |
|  | 3'-CTCCUACAAGCACUACGACUU-5' | R | 5'-CCGACTTCTTGATCTCCTCC-3' |
| SRSF11 | 5'-GGAUACCUCUAGUAAAGAATT-3' | F | 5'-CAGGAGCGAGAACCCGAG-3' |
|  | 3'-AGCCUAUGGAGAUCAUUUCUU-5' | R | 5'-CTTCTGCATATGGTACGAC-3' |
| SRSF12 | 5'-GGUAGAACAGUAUAGGUAATT-3' | F | 5'-GCCTGAGGACTTGCGCCG-3' |
|  | 3'-ACCCAUCUUGUCAUAUCCAUU-5' | R | 5'-AATCTGACGGCCACATACC-3' |
| hnRNP A2B1 | 5'-GGAUUAUUUAAUAACAUUATT-3' | F | n/a |
|  | 3'-AACCUAAUAAAUUAUUGUAAU-5' | R |  |
| hnRNP C | 5'-CGUCAGCGUGUAUCAGGAATT-3' | F | 5'-GTTACCCAGCACGTGTACC-3' |
|  | 3'-GCAGUCGCACAUAGUCCUU-5' | R | 5'-GGCCTGAAGGTCATCTCC-3' |
| hnRNP D | 5'-CAAUGUUGGUCUUAGUAAATT-3' | F | 5'-TGGGAAGGTGATTGATCC-3' |
|  | 3'-GTGUUACAACCAGAAUCAUUU-5' | R | 5'-AAGCAGAACCCACGCCTC-3' |
| hnRNP F | 5'-GCGUUCGUGCAGUUUGCCUTT-3' | F | n/a |
|  | 3'-TTCGCAAGCACGUCAAACGGA -5' | R |  |
| hnRNP H | 5'-GGUAUAUUGAAAUCUUUAATT-3' | F | n/a |
|  | 3'-GTCCAUAUAACUUUAGAAAUU-5' | R |  |
| hnRNP U | 5'-GGCCGUGGUAGUUACUCAATT-3' | F | 5'-GAGTACATTGAAGAGAACAAG-3' |
|  | 3'-GACCGGCACCAUCAAUGAGUU-5' | R | 5'-CACTGTGTCATCGAAGTGTTC-3' |
| HuR | 5'-CAGUUUCAAUGGUCAUAAATT-3' | F | 5'-ACACAGCTTGGGCTATGGC-3' |
|  | 3'-TGGUCAAAGUUACCAGUAUUU-5' | R | 5'-CATCTGAGCTCGGGCGAGC-3' |
| SMNex678 | n/a | F | 5'-CGATCTCGAGATAATTCCCCCACCACCTCCC-3' |
|  |  | R | 5'-ATATGCGGCCGCCACATACGCCTCACATACA-3' |
| SMNex45678 | n/a | F | 5'-CAGGGCCAAGACTGGGAC-3' |
|  |  | R | 5'-ATATGCGGCCGCCACATACGCCTCACATACA-3' |

**Supplementary Table 1.** siRNA duplex and primer sequences used
