# Supplementary material for: Targeting SR Proteins Improves SMN Expression in Spinal Muscular Atrophy Cells
Source: PLoS One. 2014 Dec 15;9(12):e115205. doi: 10.1371/journal.pone.0115205 (PMC4266657; doi:10.1371/journal.pone.0115205)
Supplement: S2 Table — Binding sites of proteins shown to affect SMN2 exon 7 splicing. SMN2 exon 7 and 50 nts of the upstream and downstream introns were considered for this table. A depiction of the binding sites is shown in Fig. 6. R = G or A; Y = C or U; W = A or U; N = A, G, C or U; K = U or G; S = G or C; D = A, G or U; M = A or C; H = A, C or U; V = A, C or G; B = G, C or U. (DOC) [file pone.0115205.s002.doc]

| **Splicing Factor** | **Binding Motif** | **Effect on**  **Exon 7** | **SMN2 sequence** |
| --- | --- | --- | --- |
| hnRNPA1 | RGNYAG (1-3)  GUAGUAGU (1-3)  YAGGGW(4) | Inhibitor | CAGGGU (5)  Uagaca (6, 7)  Aguaag(2, 3)  ugccag(2, 3)  Cagcauuaugaaag (8) |
| hnRNPA2B1 | GGUAGUAG (1-3)  AGGWUHGR (1-3) | Inhibitor | gguuuuag(2, 3)  cagcauuaugaaag (8) |
| hnRNPU | UGUAUUG (1-3)  YUGUKKNU (1) | Inhibitor | uauauag(2, 3)  uuuauuu(2, 3) |
| SAM68 | UUUUU(9, 10)  UAAA (10) | Inhibitor | uuuuu(11)  uuuua(12)  caaaau(11)  UUAAAU(11) |
| SRSF1 | SRSASGA(13-15)  CRSMSGW(2, 3)  UGRWGVH (2, 3) | Activator | uacaggg(16, 17)  cucacau(16, 17)  cugccag(16, 17) |
| SRSF2 | GRYYCSYR(13-15)  GRYYMCYR(2, 3)  UGCYGYY (2, 3) | Inhibitor | gucugcca(16, 17)  gaaucuua(16, 17) |
| SRSF3 | WCWWC(2, 3, 18)  CUCKUCY(2, 3, 18) | Inhibitor | acuuc(2, 3)  AUCUUAC(18) |
| SRSF5 | ACDGS(13-15)  YYWCWSG (2, 3) | Inhibitor | uuacagg(16, 17) |
| SRSF6 | HRHRKV(15, 17) | Inhibitor/  Activator | UAUAUC(16, 17)  UAUAUA(13, 15)  uaaguc(13, 15)  UGAAUC(13, 15) |
| SRSF7 | WGGACRA(2, 3)  ACGAGAGAY(2, 3) | Inhibitor | uagacaa(2, 3) |
| SRSF9 | WSSMS (19)  AGGAC (19) | Inhibitor/  Activator | AGGAA(20)  AGGAG(11) |
| TIA1 | U_(3-11)_ (21) | Activator | CUUUA(11, 22)  AUUUUC(11)  GUUUUA(11)  CUUUUG(11, 22) |
| Tra2β | GAAGAA(2, 3)  AAGUGUU(2, 3, 18)  GHVVGANR(2, 3) | Activator/ neutral | caggguu(2, 3)  aaagaa(2, 3, 23)  gaaggaag(2, 3, 23)  aggugcu(2, 3)  aagucug(2, 3, 18)  aagugaa(2, 3, 18)  AAAACUU(18)  AAACUUU(18)  AACUUUA(18) |

**Supplementary Table 2:** Binding sites of proteins shown to affect SMN2 exon 7 splicing. SMN2 exon 7 and 50nts of the upstream and downstream introns were considered for this table. A depiction of the binding sites is shown in Fig. 6. **R**=G or A; **Y**= C or U; **W**= A or U; **N**= A, G, C or U; **K**= U or G; **S**= G or C; **D**= A, G or U; **M**= A or C; **H**= A, C or U; **V**= A, C or G; **B**= G, C or U.

**Supplementary References**

1 Huelga, S.C., Vu, A.Q., Arnold, J.D., Liang, T.Y., Liu, P.P., Yan, B.Y., Donohue, J.P., Shiue, L., Hoon, S., Brenner, S. *et al.* (2012) Integrative genome-wide analysis reveals cooperative regulation of alternative splicing by hnRNP proteins. *Cell Rep*, **1**, 167-178.

2 Akerman, M., David-Eden, H., Pinter, R.Y. and Mandel-Gutfreund, Y. (2009) A computational approach for genome-wide mapping of splicing factor binding sites. *Genome Biol*, **10**, R30.

3 Paz, I., Akerman, M., Dror, I., Kosti, I. and Mandel-Gutfreund, Y. (2010) SFmap: a web server for motif analysis and prediction of splicing factor binding sites. *Nucleic Acids Res*, **38**, W281-285.

4 Burd, C.G. and Dreyfuss, G. (1994) RNA binding specificity of hnRNP A1: significance of hnRNP A1 high-affinity binding sites in pre-mRNA splicing. *Embo J*, **13**, 1197-1204.

5 Doktor, T.K., Schroeder, L.D., Vested, A., Palmfeldt, J., Andersen, H.S., Gregersen, N. and Andresen, B.S. (2011) SMN2 exon 7 splicing is inhibited by binding of hnRNP A1 to a common ESS motif that spans the 3' splice site. *Hum Mutat*, **32**, 220-230.

6 Cartegni, L., Hastings, M.L., Calarco, J.A., de Stanchina, E. and Krainer, A.R. (2006) Determinants of exon 7 splicing in the spinal muscular atrophy genes, SMN1 and SMN2. *Am J Hum Genet*, **78**, 63-77.

7 Kashima, T. and Manley, J.L. (2003) A negative element in SMN2 exon 7 inhibits splicing in spinal muscular atrophy. *Nat Genet*, **34**, 460-463.

8 Hua, Y., Vickers, T.A., Okunola, H.L., Bennett, C.F. and Krainer, A.R. (2008) Antisense masking of an hnRNP A1/A2 intronic splicing silencer corrects SMN2 splicing in transgenic mice. *Am J Hum Genet*, **82**, 834-848.

9 Itoh, M., Haga, I., Li, Q.H. and Fujisawa, J. (2002) Identification of cellular mRNA targets for RNA-binding protein Sam68. *Nucleic Acids Res*, **30**, 5452-5464.

10 Lin, Q., Taylor, S.J. and Shalloway, D. (1997) Specificity and determinants of Sam68 RNA binding. Implications for the biological function of K homology domains. *J Biol Chem*, **272**, 27274-27280.

11 Piva, F., Giulietti, M., Burini, A.B. and Principato, G. (2012) SpliceAid 2: a database of human splicing factors expression data and RNA target motifs. *Hum Mutat*, **33**, 81-85.

12 Pedrotti, S., Bielli, P., Paronetto, M.P., Ciccosanti, F., Fimia, G.M., Stamm, S., Manley, J.L. and Sette, C. (2010) The splicing regulator Sam68 binds to a novel exonic splicing silencer and functions in SMN2 alternative splicing in spinal muscular atrophy. *Embo J*, **29**, 1235-1247.

13 Cartegni, L., Wang, J., Zhu, Z., Zhang, M.Q. and Krainer, A.R. (2003) ESEfinder: A web resource to identify exonic splicing enhancers. *Nucleic Acids Res*, **31**, 3568-3571.

14 Liu, H.X., Zhang, M. and Krainer, A.R. (1998) Identification of functional exonic splicing enhancer motifs recognized by individual SR proteins. *Genes Dev*, **12**, 1998-2012.

15 Smith, P.J., Zhang, C., Wang, J., Chew, S.L., Zhang, M.Q. and Krainer, A.R. (2006) An increased specificity score matrix for the prediction of SF2/ASF-specific exonic splicing enhancers. *Hum Mol Genet*, **15**, 2490-2508.

16 Smith, P.J., Zhang, C., Wang, J., Chew, S.L., Zhang, M.Q. and Krainer, A.R. (2006) An increased specificity score matrix for the prediction of SF2/ASF-specific exonic splicing enhancers. *Hum Mol Genet*, **15**, 2490-2508.

17 Cartegni, L., Wang, J., Zhu, Z., Zhang, M.Q. and Krainer, A.R. (2003) ESEfinder: A web resource to identify exonic splicing enhancers. *Nucleic Acids Res*, **31**, 3568-3571.

18 Paz, I., Kosti, I., Ares, M., Jr., Cline, M. and Mandel-Gutfreund, Y. (2014) RBPmap: a web server for mapping binding sites of RNA-binding proteins. *Nucleic Acids Res*, in press.

19 Paradis, C., Cloutier, P., Shkreta, L., Toutant, J., Klarskov, K. and Chabot, B. (2007) hnRNP I/PTB can antagonize the splicing repressor activity of SRp30c. *Rna*, **13**, 1287-1300.

20 Young, P.J., DiDonato, C.J., Hu, D., Kothary, R., Androphy, E.J. and Lorson, C.L. (2002) SRp30c-dependent stimulation of survival motor neuron (SMN) exon 7 inclusion is facilitated by a direct interaction with hTra2 beta 1. *Hum Mol Genet*, **11**, 577-587.

21 Dember, L.M., Kim, N.D., Liu, K.Q. and Anderson, P. (1996) Individual RNA recognition motifs of TIA-1 and TIAR have different RNA binding specificities. *J Biol Chem*, **271**, 2783-2788.

22 Singh, N.N., Seo, J., Ottesen, E.W., Shishimorova, M., Bhattacharya, D. and Singh, R.N. (2011) TIA1 prevents skipping of a critical exon associated with spinal muscular atrophy. *Mol Cell Biol*, **31**, 935-954.

23 Hofmann, Y., Lorson, C.L., Stamm, S., Androphy, E.J. and Wirth, B. (2000) Htra2-beta 1 stimulates an exonic splicing enhancer and can restore full-length SMN expression to survival motor neuron 2 (SMN2). *Proceedings of the National Academy of Sciences of the United States of America*, **97**, 9618-9623.
